# Constructing a dual-factor, dual-pathway framework for analyzing parent–child interactions: an exploration of parental insightfulness and beliefs

**DOI:** 10.3389/fpsyg.2026.1783663

**Published:** 2026-03-26

**Authors:** Zihan Guo, Lei Wang, Yonggang Ren

**Affiliations:** 1 College of Child Development and Education, Zhejiang Normal University, Zhejiang, China; 2 School of Education, University of New England, Armidale, NSW, Australia

**Keywords:** dual-pathway framework, parental beliefs, parental insightfulness, parent–child interactions, toddler

## Abstract

Current research on parent “child interaction focuses primarily on parents” observable characteristics with limited exploration of the underlying psychological processes driving interactive behaviors. This study explores an implicit process model of parent-child interactions through two factors parental insightfulness, which is defined as parents' ability to receive and interpret children's signals, and parental beliefs. Using video-stimulated recall interviews and turn-by-turn microanalysis with 35 parent-child dyads (children aged 12 to 36 months), two psychological pathways were identified based on dual-process models of information processing: The Parental Insightfulness-dominant Pathway (child's signal → parental reception and interpretation → responsive behavior) and the Parental Beliefs dominant Pathway (parental beliefs → interactive behavior). The research findings indicate that in the total of 223 interactions parents were more inclined to use the Parental Insightfulness-dominant Pathway rather than the Parental Beliefs-dominant Pathway. Furthermore, in the Parental Insightfulness-dominant Pathway, if parents can accurately interpret the signals, they are more likely to respond appropriately. However, in Parental Beliefs-dominant Pathway, parents often initiate or guide interactions based on their own parental beliefs, leading to inappropriate behavioral patterns. Our qualitative analyses demonstrated that parental insightfulness mediated between parents' interpreting children's signals and their responses and hence improved the quality of interactions. Therefore, it is essential to cultivate parental insightfulness based on scientific parental beliefs, employing a dual- factor approach to optimize the practice of responsive caregiving in early childhood development.

## 1 Introduction

Family constitutes the primary context for early childhood development. The quality of parent–child interactions exerts a profound influence on multiple dimensions of physical and psychological growth during the first few years of life. Parent–child interactions have been investigated extensively, with majority focusing on interaction types (e.g., high-control/compliance and low-interaction), behavioral characteristics (e.g., sensitivity and responsiveness), quality (e.g., fathers tend to ask open-ended questions more frequently than mothers, and these questions are more effective at fostering children's exploratory dialogue), and reduced interactional synchrony in families where children had additional needs (Zheng and Chen, 2003; Bornstein and Manian, 2013; Leech et al., 2023; Del Rosario et al., 2023). These studies employed standardized tools such as *Caregiver Interaction Scale* (CIS) and *Parenting Interactions with Children: Checklist of Observations Linked to Outcomes* (PICCOLO), and used a range of methods including questionnaires such as the Brigance Parent–Child Interactions Scale (BPCIS), laboratory observations (such as free play or puzzle tasks), and naturalistic video recordings (Colwell et al., 2013; Roggman et al., 2013; Glascoe, 2002). However, most existing research focuses on examining parent–child interactions through overt behaviors, with little in-depth exploration of the underlying psychological mechanisms behind these interactions (List et al., 2021; Roubinov and Boyce, 2017).

Current research categorizes the factors shaping parent–child interactions into three domains: child characteristics (e.g., temperament, gender, physical and mental health), parental characteristics (e.g., parental style, socioeconomic status, mental health, family relationships and social networks), and environmental influences (e.g., cultural traditions, and digital media exposure) (Kirchhoff et al., 2019; Clearfield and Nelson, 2006; Hall et al., 2015; Jiang et al., 2023; Gago-Galvagno and Elgier, 2020; Brookman et al., 2023; Cochran and Niego, 2002; Kildare and Middlemiss, 2017; Lansford, 2022; Stroud et al., 2011). Among these, parental characteristics is the most investigated factor (Mountain et al., 2017). Research has identified correlations between observable factors and parent–child interactions (Gago-Galvagno and Elgier, 2020; Jennings et al., 1991), and findings suggest that some unobserved factors, such as parental beliefs, may also have impacts on parent–child interactions (Sigel, 2014).

Parental beliefs, typically conceptualized as relatively stable cognitive frameworks, have not been sufficiently examined with regard to how they operate through implicit process model during parents' real-time interactions with their children. Although previous studies have highlighted parental insightfulness—the key link between parents' interpretation of child signals and their behavioral responses—as a critical factor influencing interaction quality, the underlying mechanisms through which this process unfolds remain insufficiently understood (Marcu et al., 2016; Oppenheim et al., 2023). Moreover, while parental insightfulness and beliefs have each been investigated separatel, their interrelationship, as well as their potential synergistic effects within the parent–child interaction process, has yet to be empirically tested. The parent–child interaction process generally comprises three interlinked steps: sensitive attention, accurate interpretation, and appropriate responding, with responsive caregiving being a critical component of the Nurturing Care Framework (World Health Organization, 2018). This framework provides important insights for exploring the implicit process model through which parental insightfulness and beliefs operate in parent–child interactions. Based on research findings and analysis, this study intends to offer specific suggestions to enhance parental insightfulness, optimize parental beliefs, strengthen parent–child relationships, and support families in fostering appropriate parenting environments for optimal child development.

## 2 Literature review

### 2.1 Key factors influencing parent–child interactions

Early childhood life primarily takes place at home, where the quality of parenting is closely correlated with the development of children aged 0–3 years. Extensive research shows that positive interactive stimuli, including eye contact, physical touch, and verbal/non-verbal communication between caregivers and children, can alter brain structures and promote early development (Feldman et al., 2013; Rhoades et al., 2011). Parents serve as children's primary caregivers, initiating and facilitating interactions. Therefore, focusing on parental traits is an effective approach to pinpoint avenues for enhancing interaction quality (Mountain et al., 2017). From a parental perspective, key influencing factors include parental beliefs (e.g., views of the child, educational values, and parenting knowledge), demographic variables (e.g., family socioeconomic status), and other contextual factors (e.g., parental physical and mental health, and family relationships) (Schofield and Weaver, 2016; Grose et al., 2024; Huang et al., 2005; Gago-Galvagno and Elgier, 2020; Brookman et al., 2023; Stroud et al., 2011). Empirical evidence suggests that these factors influence parent–child interaction behaviors primarily through parental beliefs as a mediating mechanism (Roubinov and Boyce, 2017; Xin and Yu, 2024). In addition, parents can only respond appropriately and foster constructive interactions when they accurately understand the child's needs (Leerkes, 2010). This capability is termed “sensitivity” and conceptualized as “parental insightfulness” (Ainsworth and Bell, 1970; Koren-Karie and Oppenheim, 2018).

### 2.2 Definition of parental insightfulness and its measurement

Parental insightfulness is the ability to perceive situations from the child's perspective and infer the underlying motivations and emotions that influence the child's conduct (Koren-Karie et al., 2002). This manifests as the parent's ability to attribute intrinsic meaning to the child's overt behaviors (Feniger-Schaal and Koren-Karie, 2021). The concept has significant theoretical overlaps with maternal sensitivity and mind-mindedness. Specifically, maternal sensitivity entails accurately recognizing and interpreting children's signals, forming motivational orientations, and selecting appropriate behavioral responses (Larkin et al., 2021). Mind-mindedness denotes the parent's capability to view the child as an independent individual with their own mind and describe the child's mental states spontaneously during interactions (Meins, 1997). Mentalization refers to parents' ability to understand and perceive their own and their children's mental states, and this capacity facilitates the formation of attachment relationships (Fonagy, 1991). While related to these concepts, parental insightfulness specifically emphasizes the depth of the parent's understanding of the child's distinct inner world (Koren-Karie et al., 2002; Feniger-Schaal and Koren-Karie, 2021).

Parent–child clinicians and developmental researchers argue that a comprehensive understanding of parent–child interactions, particularly within distressed, conflictual, or non-harmonious relationships, requires an enhanced comprehension of the child's inner world (Koren-Karie and Oppenheim, 2018). Systematic approaches have been developed to assess caregivers' insights into parenting. For instance, Adult Attachment Interview (AAI) evaluates attachment related internal working models by analyzing parents' narratives of their own developmental experiences and emotional histories, thereby providing a foundation for understanding parental psychological structures (Main et al., 1985). Working Model of the Child Interview (WMCI) examines the depth of parents' understanding of their child's traits, behaviors, and emotional responses, reflecting how parents apply their own mental representations to the construction of their child's mental representations (Zeanah et al., 1994). The Parent Development Interview (PDI) emphasizes parents' overall understanding of the parent–child relationship, encompassing role perceptions and emotional experiences, reflecting the integration and application of mental representations at the relational level (Slade et al., 2003). Finally, the newly developed Insightfulness Assessment (IA) Scale employs a video-stimulated recall interview system to directly evaluate parents' psychological capacity to understand young children's perspectives.

### 2.3 The relationship between parental insightfulness and parent–child interactions

Responsive Caregiving posits that high-quality parent–child interaction requires caregivers to closely observe children's signals—such as body movements, vocalizations, and gestures—and respond promptly and appropriately to their needs through physical contact, eye contact, facial expressions, and verbal communication (World Health Organization, 2018). Zhang and Zhou (2022) summarized three key characteristics of responsive interaction: sensitive attention, accurate interpretation, and appropriate response corresponds to parent–child interaction behaviors. Parental insightfulness focuses on the internal processes of parenting, whereas parent–child interaction behaviors represent the external manifestations of the parenting process (Zhang and Zhou, 2022).

Extensive empirical research supports a positive association between parental insightfulness and sensitive interaction. Parents with high insightfulness misinterpret children's signals less frequently and provide more synchronized, supportive care, whereas low insightfulness is often linked to controlling, directive, or even intrusive interaction patterns (Koren-Karie et al., 2002; Suardi et al., 2017). Moreover, mothers who demonstrate proactive parental insightfulness exhibit greater synchrony and sensitivity in parent–child interactions (Feniger-Schaal et al., 2019).

Furthermore, parental insightfulness acts as a buffer against adverse life events. While postpartum stressful events predicted fewer positive parent–child interactions among mothers with non-positive insightfulness, this negative effect was absent in mothers with positive insightfulness (Martinez-Torteya et al., 2018). Intervention studies also show that parenting quality improvements are more pronounced among mothers with positive insightfulness (Siller et al., 2018). Additionally, examining the impact of parental insightfulness on family nurturing patterns within a triadic family interaction model (father, mother, and child) revealed that: Families where both parents exhibit positive parental insightfulness demonstrate greater ability to understand and respond to the child's emotions and needs, thereby fostering more flexible, synchronized, and coordinated interactions among the three participants (Marcu et al., 2016).

### 2.4 The impact of parenting beliefs on parental insightfulness and interactive behaviors

Parental beliefs typically encompass two core dimensions: first, fundamental assumptions about the nature and developmental patterns of children (view of the child); second, the conceptual framework governing educational goals and parenting approaches (view of education). This distinction has been emphasized in numerous studies examining parental belief systems (Palacios and Moya, 1990; Murphey and Martin, 1992; Sigel, 1985).

Firstly, parental beliefs directly influence caregivers' interaction styles. For instance, how historical family interactions shaped by parental beliefs impact current parent–child interactions. Existing research indicates that shared parenting patterns formed during pregnancy become a “template” for subsequent parent–child interactions. Even when environmental changes occur after the child's birth, this interaction structure remains stable (Favez et al., 2006). Secondly, parental beliefs directly influence parents' sensitivity to and understanding of children's signals. When parents hold beliefs oriented toward empathy, developmentally appropriate responses, and respect for the child's individuality, they are more likely to interpret the child's behaviors and expressions as meaningful signals of emotion or intent. They then respond in sensitive and supportive ways, significantly enhancing the quality of parent–child interactions (Slade, 2005; Dix, 2000). Conversely, if parents' parenting approach leans toward authoritarianism or emphasizes obedience, they are more likely to interpret children's behavior as uncooperative or defiant, thereby overlooking the child's genuine emotions and psychological needs (Goagoses et al., 2023). Such misinterpretations undermine their ability to discern the child's inner state, leading to more controlling and less responsive interactions that diminish engagement quality. Furthermore, parenting philosophies indirectly shape parental interaction styles through perceptual frameworks. Non-intrusive parenting emphasizes child-centered approaches, acknowledging and respecting the validity of the child's perspective. This enables accurate interpretation of the child's signals and prevents parental goals from dominating activities. In contrast, intrusive parenting centers interactions around adult agendas and control, often overlooking the child's immediate needs and cues (Fuligni and Brooks-Gunn, 2013).

### 2.5 The present study

Current mainstream research on parent–child interaction focuses primarily on parents' observable characteristics (such as educational attainment, parenting styles, and caregiver stress), with limited exploration of the underlying psychological processes driving interactive behaviors. Furthermore, parental insightfulness has received relatively less attention, with existing studies covering its influencing factors, relationships with attachment and sensitivity, early childhood development, and intervention strategies targeting parental insight. Although numerous studies exist on parental beliefs about childrearing, the implicit mechanisms through which these beliefs influence parental insightfulness and subsequently shape parent–child interaction behaviors remain under explored. Therefore, this study poses the following questions:

1. What implicit psychological pathways do parental insightfulness and beliefs operate through in parent–child interactions, and how do they function?
2. What preferences do parents exhibit in these implicit psychological pathways during interactions, and what corresponding effects do these interactions produce?

## 3 Methods

### 3.1 Participants

The participants comprised 35 toddlers aged from 12 to 36 months with an average age of 26.34 months. There were 18 boys (51.4%) and 17 girls (48.6%), and 19 (54.3%) toddlers were the only children of the families. Among the 35 parent participants, 31 (88.6%) were mothers and 4 (11.4%) were fathers. Twenty-seven (77.2%) parents held a bachelor's degree or higher, and the remaining 8 (22.8%) parents had high school graduation certificates or equivalents. Twenty-seven (77.2%) parents reported a monthly household income exceeding ¥20,000 RMB (US$2791), 5 (14.3%) parents had a monthly income ranging from 10,001 to 20,000 RMB (US$1395–2791), and the remaining 3 (8.5%) parents had a monthly income below 10,000 RMB (US$1395). Regarding occupations, 16 (45.7%) parents stated they were semiprofessionals or public servants, 8 (22.8%) were academics or government officials, 6 (17.2%) were tradespeople or small business owners, and 5 (14.3%) were un-employed or doing low-skilled work.

### 3.2 Procedures

Thirty-five children were selected from two types of early childhood education institutions: private childcare centers and public childcare centers. All the childcare centers were located in Hangzhou City, Zhejiang Province in Eastern China. Prior to the child recruitment, their parents received invitation letters from researchers, with participant information and consent forms included. The parent–toddler interactions of all the participants were video recorded in a research laboratory at Zhejiang Normal University. After collecting the videos, the researchers used the first 2 min of the recordings as a stimulation prompt and conducted semi-structured interviews with the parents to explore their interpretations of their children's thoughts and feelings during their interactions with their children. In the interview verbatim transcripts, all the participant personal identifications were removed to protect their privacy. When all the data were collected, the parents were provided with free parental insightfulness training, and the parents and their children were rewarded with a book and a toy. Ethics approval was obtained through an extensive process at the authors' institution (Approval No. ZSJR2419004), and participation was entirely voluntary.

### 3.3 Measures

#### 3.3.1 Insightfulness assessment (IA)

Firstly, the parent–child interactions were video recorded in three scenarios: free play (20 min), daily caregiving (8 min), and a parental distraction task (8 min). The entire recording lasted 36 min for each parent–child dyad. During the free-play session, the children and the parents were given three bags filled with age-appropriate toys to play with. The second session was caregiving, where the parents fed the toddlers, changed their diapers, and dressed the children. The third one was the parent distraction task, where the children played with toys while their parents filled out a form. The third session was excluded from the subsequent qualitative analyses because there were very few full rounds of parent–child interactions and provided limited value to investigate how parental insightfulness worked.

The Insightfulness Assessment (IA) (Koren-Karie et al., 2002) was used as a guide for the researchers to interview the parents. The first 2-min chunks of the recordings from the first two sessions, namely the free play and the caregiving sessions, were played to the parents. The listed questions were asked to the parents in the following video-stimulated recall interview:

1. In your opinion, what thoughts and feelings did your child have during this session?
2. Did your child generally exhibit these behavioral characteristics as we observed in the video?
3. How did you feel when you were watching the video? Did anything surprise you, worry you, or make you happy?

Two general questions were asked to the parents after the interview with regards to each of the three sessions:

1. What do you like the most about your child based on what we have seen today and your general understanding of them?
2. After sharing your feelings regarding each session, is there anything else about your child in your daily life that surprises you, concerns you, or makes you happy?

Throughout the interview, the parents were asked to support their claims with examples from their observations of their children. All identifying information concerning parents and their children (e.g., names) has been removed from the interview transcripts. It should be noted that this study did not adhere to the instrument's original protocol for quantifying parental insightfulness scores as an independent psychometric measurement instrument. Instead, parental insightfulness interviews based on IA interview guide were employed as a qualitative data-elicitation method to access parents' understanding of their children's inner worlds and their parental beliefs.

#### 3.3.2 Turn-by-turn

Inspired by the serve-and-return model proposed by the Harvard Centre, this study further operationalizes this dynamic interaction perspective into a micro-level analytical framework, introducing a turn-by-turn analysis methodology (National Scientific Council on the Developing Child, 2004). This study hypothesizes that each round of parent–child interaction corresponds to an implicit information-processing cycle. The interaction cycle commences with a signal initiated by the child and concludes with the parent's response, with the “turn” serving as the fundamental unit of analysis. Specifically, the free play session consisted of three settings: a role play in the kitchen, a doctor role play, and shared picture book reading. In the free play scenario, 35 parent–child pairs completed a total of 119 interaction turns. Daily care activities yielded 104 complete interaction turns from the 35 parent–child pairs. Therefore, the total sum of 223 interactive turns was elicited from the first 2 min of parent–child interactions in the free play and the daily caregiving contexts.

## 4 Coding framework

### 4.1 Theoretical foundation

**Figure 1:**
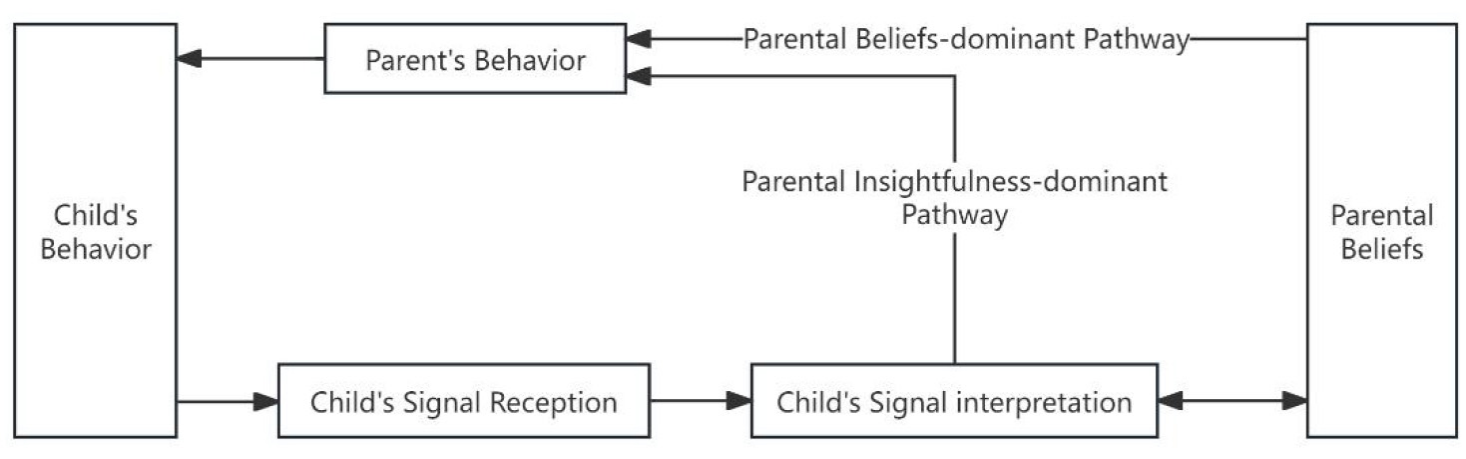
Theoretical conceptualization of implicit information processing pathways in parent–child interaction.

The dual-process models of information processing states that information processing involves two pathways: the bottom-up “data-driven” pathway, wherein the human brain perceives and registers external stimuli, engages in working memory, facilitates interactions between long-term and working memory, and ultimately generates responses; and the top-down “concept-driven” pathway, wherein external stimuli automatically activate information stored in long-term memory, directly eliciting responses (Petty and Cacioppo, 1986). Through theoretical deduction, it can be inferred that the information processing involved in parent–child interactions may also follow two distinct pathways. The first pathway comprises the following steps: the child sends signals, the parent receives signals, the parent interprets the signals based on working memory and long-term memory interactions, and the parent implements interactive strategies and behaviors. The process of “receiving and interpreting” the child's behavioral signals can be collectively termed “parental insightfulness” (World Health Organization, 2018). This pathway may be termed the “parental insightfulness-driven pathway.” The second pathway involves the following sequence: the child sends signals, parents receive these signals, which automatically activate parents' long-term memory of parental beliefs (such as perceptions of the child, parenting expectations, and interaction strategies), and parents then employ interaction strategies and behaviors. This pathway may be termed the “parental belief-driven pathway.” See Figure 1.

### 4.2 Coding framework description

Based on the theoretical conceptual model outlined above, each interaction cycle comprises elements such as the child's behavioral signals, the parent's reception and interpretation of these signals, the parent's parental beliefs, and the parent's interactive behaviors. As parent–child interactions continue, these elements are cyclically repeated. Therefore, this study adopts a single interaction cycle as the minimal unit of analysis (sample), forming the basic framework for qualitative coding from the aforementioned elements. During actual coding, researchers cross-referenced parental interview data with parent–child interaction video materials to progressively refine the coding framework. Ultimately, the parental insightfulness dimension was expanded into three categories: “*No Parental Insightfulness*,” “*Accurate Parental Insightfulness*,” and “*Biased Parental Insightfulness*.” The parental interactive behavior dimension was refined into two categories: “*Appropriate Behavior*” and “*Inappropriate Behavior*.”

To be specific, first, the children's needs, intentions, and emotions were identified based on their signals, including gaze, pointing behaviors, and verbal expressions. If the parents fail to follow the children's points of interest, do not establish joint attention, or if the parents persist with their own parenting goals without considering the children's signals—these situations can be classified as “*No Parental Insightfulness”*. Second, we cross-referenced parents' interview-based interpretations of their children's internal status with the children's behaviors observed in video recordings. When parents” interpretations were consistent with the children's observed behaviors, this can be classified as “*Accurate Parental Insightfulness”*; when discrepancies were evident, it can be classified as“* Biased Parental Insightfulness”*. For example, one father mentioned in an interview: “My child usually prefers novel toys and switches to a new one after playing briefly with any toy.” This understanding of his child aligns with the child's behavior in the video: the child is playing with a medical kit, fiddles with the stethoscope briefly before discarding it, then continues searching the kit for medicine bottles, shakes a bottle a few times, and then starts looking for a syringe. Third, when parents initiated or responsive actions meet the child's needs and intentions, resulting in emotional stability or joy; or when parents assist the child in resolving a problem without taking over, their behavior can be deemed “*appropriate behavior”* Conversely, “*inappropriate behavior”* occurs when parents fail to respond to the child's signals. For example, a father ignores his child's nonverbal signals, such as pleading looks, for a new toy and instead turns to other activities. Another example is while the child is focused on eating fruit, the mother abruptly demands the child identify and count the fruits in front of them, persistently asking: “Baby, what kind of fruit are you eating?” “How many fruits do you see in front of you?”

Through coding analysis, the parental insightfulness-dominated pathway can be subdivided into three sub-pathways: On Sub-pathway 1, parents are sensitive to children's behavioral signals, interpret them accurately, and engage in appropriate interactions. On Sub-pathway 2, parents are sensitive to children's behavioral signals and interpret them accurately, but their interactions are inappropriate. On Sub-pathway 3, parents are sensitive to children's behavioral signals but interpret them biased and engage in inappropriate interactions. The parental beliefs-dominant pathway can be subdivided into two sub-pathways: On Sub-pathway 4, parents act based on their parental beliefs but engage in inappropriate interactions; on Sub-pathway 5, parents act based on their parental beliefs and engage in appropriate interactions.

### 4.3 Intercoder reliability

All the data were coded by two research teams, with each team comprising a principal investigator (1st and 2nd authors respectively) and a student undertaking a post-graduate course in early childhood education. Initially, the first 20% of the parent–child interactions (45 turns) were coded independently by the two teams simultaneously. After achieving an agreement of 80% of the analyzed codes, the two teams proceeded to code the remaining 80% of parent–child interactions (178 turns) separately. The overall Kendall's W coefficients for the two teams were 0.900 and 0.919, indicating a high level of coding consistency (Kendall and Smith, 1939).

## 5 Results

### 5.1 How parental insightfulness and beliefs influenced parent–child interactions: quantitative analyses

Table 1 presents the distribution of parental reception, interpretation, and response to children's signals across 223 parent–child interaction tunes in free play and daily care-giving. Based on the distribution and combination of these data, the subsequent distribution of two major psychological pathways and five sub-pathways of parental cognitive processing emerged.

**Table 1:** Parental responses to child signals during free play and daily caregiving.

**Child's signal receptions**	**Received**			**Ignored**		**Sum**
**Child's signal interpretation**	**Accurate**		**Biased**		
**Parents' behavior**	**Appropriate sub-pathway 1**	**Inappropriate sub-pathway 2**	**Inappropriate sub-pathway 3**	**Appropriate sub-pathway 4**	**Inappropriate sub-pathway 5**	
Free play	43	20	3	16	37	119
Daily caregiving	50	16	1	9	28	104
Sum	93	36	4	25	65	223

In free play scenarios, 66 times (55.5%, comprising 43 Sub-pathway 1 + 20 Sub-pathway 2 + 3 Sub-pathway 3) of children's signals were received, while 53 times (44.5%, comprising 16 Sub-pathway 4 + 37 Sub-pathway 5) were ignored. Among the 66 received signals, 63 times (95.5%, comprising 43 Sub-pathway 1 + 20 Sub-pathway 2) were accurately interpreted; only 3 times (4.5%) showed biased interpretation (Sub-pathway 3). Regarding parental responses, 59 times (45.0%, comprising 43 Sub-pathway 1 + 16 Sub-pathway 4) were deemed appropriate, while 60 times (55.0%, comprising 20 Sub-pathway 2 + 3 Sub-pathway 3 + 37 Sub-pathway 5) were inappropriate. In daily care scenarios, 67 signals (64.4%, comprising 50 Sub-pathway 1 + 16 Sub-pathway 2 + 1 Sub-pathway 3) were received, while 37 (35.6%, comprising 9 Sub-pathway 4+ 28 Sub-pathway 5) were ignored. Among the 67 signals received, 66 (98.5%, comprising 50 Sub-pathway 1 + 16 Sub-pathway 2) were accurately interpreted, with only 1 (1.5%) showing biased interpretation (Sub-pathway 3). Regarding parental responses, 59 times (56.7%; 50 Sub-pathway 1 + 9 Sub-pathway 4) were deemed appropriate, while 45 (43.3%; 16 Sub-pathway 2 + 1 Sub-pathway 3 + 28 Sub-pathway 5) were identified as inappropriate.

Table 2 reveals notable differences between Parental Insightfulness-dominant Pathway (data-driven) and Parental Beliefs-dominant Pathway (concept-driven) (59.6% vs. 40.5%), with the parents showing a greater tendency to choose Parental Insightful-ness-dominant Pathway. This indicates that the parents were more likely to perceive the children's immediate needs before responding to them.

**Table 2:** Comparison of two psychological pathways.

**Pathway**	**Parental insightfulness-dominant pathway**	**Parental beliefs-dominant pathway**	**Sum**
*N* (%)	133 (59.6%)	90 (40.4%)	223 (100%)

As indicated in Table 3, within Parental Insightfulness-dominant Pathway, Sub-pathway 1 exhibited the highest proportion (69.9%) and exceeded the combined total of Sub-pathway 2 (27.1%) and Sub-pathway 3 (3.0%). This finding suggests that most parents could sensitively receive their children's signals and accurately interpret them. They subsequently integrated appropriate interactive strategies from their parental beliefs and initiated appropriate responses, facilitating high-quality parent–child interactions. Sub-pathway 1 and Sub-pathway 2 together amounted to 97% in Parental Insightfulness-dominant Pathway. This result suggests that the majority of parents were able to accurately interpret the children's signals and draw reasonable conclusions about the children's underlying intentions. However, the use of unsuitable interactive strategies still led to inappropriate responses as Sub-pathway 2 shows. Within Parental Beliefs-dominant Pathway, Sub-pathway 4 (72.2%) was significantly higher than Sub-pathway 5 (27.8%). This indicates that interactions initiated from parental beliefs were more likely to result in inappropriate interactive behaviors.

**Table 3:** Five sub-pathways under two psychological pathways.

**Pathway**	**Parental insightfulness-dominant pathway**			**Parental beliefs-dominant pathway**	
**Sub-pathway**	**Sub-pathway 1**	**Sub-pathway 2**	**Sub-pathway 3**	**Sub-pathway 4**	**Sub-pathway 5**
*n* (%)	93 (69.9%)	36 (27.1%)	4 (3.0%)	65 (72.2%)	25 (27.8%)
*N* (%)	133 (100%)			90 (100%)	

As indicated in Table 4, Sub-pathway 1 (41.7%) and Sub-pathway 4 (29.1%) exhibited the higher frequencies during the interactions, followed by Sub-pathway 2 (16.1%) and Sub-pathway 5 (11.8%), while Sub-pathway 3 (1.3%) was the least common.

**Table 4:** Parents' preferences across five sub-pathways.

**Sub-pathway**	**Sub-pathway 1**	**Sub-pathway 2**	**Sub-pathway 3**	**Sub-pathway 4**	**Sub-pathway 5**	**Sum**
*n* (%)	93 (41.7%)	36 (16.1%)	4 (1.3%)	65 (29.1%)	25 (11.8%)	223 (100%)

### 5.2 How parental insightfulness and beliefs influenced parent–child interactions: qualitative analyses

In this section, typical cases from the free play and the daily caregiving sessions were used to illustrate the presence of all the five interaction sub-pathways. The qualitative analyses intended to unfold the crucial mediating role of parental insightfulness in parent–child interactions.

#### 5.2.1 Sub-pathway 1 in free play

**Figure 2:**
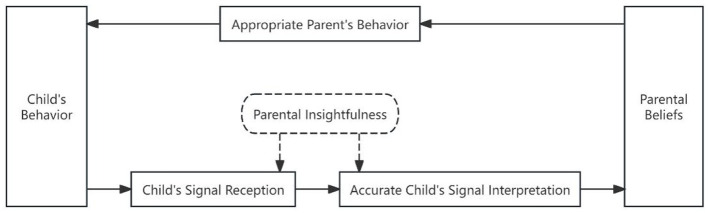
Sub-pathway 1 in free play.

Figure 2 presents that, within Sub-pathway 1 of Psychological Pathway One, parents receive children's signals and interpret them accurately based on their parenting beliefs in long-term memory. They select suitable interactive strategies and respond with appropriate behaviors.

Vignette 1: Niuniu (male, 21 months) and his father (31 years) were playing block construction together at a long table. Suddenly, Niuniu raised his hand and pointed to a small medical kit on the other side of the table. The father asked, “Do you prefer to play with the bag?” The child nodded for “yes”. Grabbing the medical kit, the father proposed, “Let's tidy up the blocks first.” He began to pack up the scattered blocks, but Niuniu did not tidy up. Instead, he showed much interest in the medical kit, opened it and explored its contents. When the father saw this, he stopped packing up, cleared the table, and immediately joined the exploratory activity with the child.

Analysis: When the child Niuniu lost interest in the blocks, he pointed to the medical kit (child's signal), and the father promptly received the signal. He verified the child's desire by asking whether he wanted to play with the medical kit (parent's behavior). This aligns perfectly with what the father expressed in the interview: “[Niuniu] lost interest in the blocks” (parental insightfulness) and “I usually observe what captures his interest first. If he wants to switch toys, I follow his lead and don't force him back to the original activity” (parent's view of education). During this turn of parent–child interaction, the child's signal was clear and the father made reasonable response based on the child's body language and responded appropriately. The child's swift engagement in the new game afterward indicates that the father's response aligned with the child's current needs.

#### 5.2.2 Sub-pathway 2 in daily caregiving

**Figure 3:**
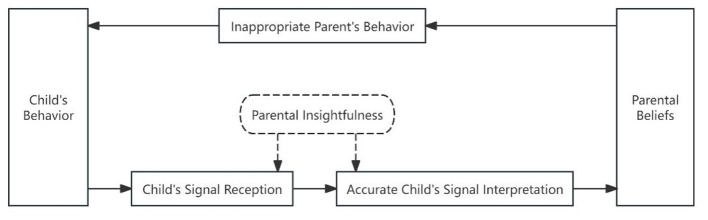
Sub-pathway 2 in daily caregiving.

Figure 3 presents that, within Sub-pathway 2 of Psychological Pathway One, parents receive children's signals and interpret them accurately based on parenting beliefs in long-term memory. However, they select unsuitable interactive strategies and respond with inappropriate behaviors.

Vignette 2: Momo (male, 34 months) was eating an apple when the father (27 years) asked, “Can I have a bite?” Momo immediately moved the apple toward his father's mouth. The father pretended to bite it while producing chewing sounds (“Mm-ah”), which made the child delighted with laughter. Then Momo stood up holding the apple high and said, “I'll put it in your mouth!” The father repeated the child's utterance, “You'll put it in my mouth? No thanks.” and prompted the child to return to his seat and keep eating.

Analysis: The father's interview statement, “(Momo) enjoys food-sharing” (parental insightfulness). He also believes that MoMo has become a “child with his own thoughts who is willing to actively interact with others,” reflecting the father's overall approach of viewing young children as subjects with emotions and intentions (parent's view of the child). In actual interaction, the child promptly shared his fruit with the father after he made a request, maintaining a cheerful mood throughout (child's signal). This indicates that the father's insight into the child's current thoughts and feelings is accurate. Nevertheless, he asked the child to share the fruit but refused after the child expressed a willingness and further action to share (parent's behavior). It is likely that the father solely wanted to test whether the child had willingness to share, therefore his final response was inconsistent with his initial request. This is not positive behavior modeling as it could diminish the child's enthusiasm for sharing.

#### 5.2.3 Sub-pathway 3 in free play

**Figure 4:**
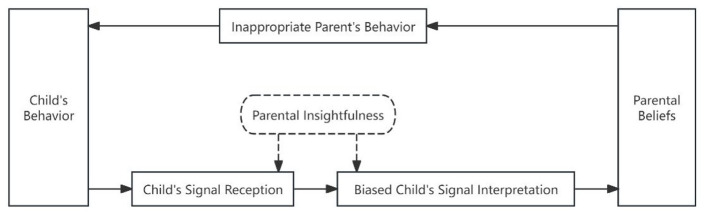
Sub-pathway 3 in free play.

Figure 4 presents that, within Sub-pathway 3 of Psychological Pathway One, parents receive children's signals and interpret these signals with bias based on relevant parenting beliefs in long-term memory. They then select inappropriate interactive strategies, resulting in inappropriate responses.

Vignette 3: Qiuqiu (male, 22 months) and his father (31 years) were pretending cooking in a kitchen. The boy saw a lavalier microphone attached to his father's collar and looked closely at it. The father asked, “What are you looking at?”, took off the device, and held it in his hands. Qiuqiu became more curious and approached closer to have a look, but his father continued to play with kitchenware. The child reached for the microphone, but the father replied, “Don't touch it. I won't let you take it. You can take this little teapot.” He moved Qiuqiu away from the microphone and then used a toy to distract his attention.

Analysis: In the interview, the father said “These are new toys that he never played with at home (parental insightfulness), and he likes new toys (parent's view of child).” However, our observation shows that Qiuqiu was not keen on the new toys but showed interest in the microphone that suddenly appeared in his father's collar. His eyes fixed on the recording device and his hand reaching out to grab the microphone (child's signal). Although the father received the signal, he interpreted the signal in a biased way (a minor incident not worth dwelling on). Consequently, the father's focus remained fixed on the toy at hand, using it to divert the child's attention (parent's behavior). He demonstrated insufficient sensitivity to the “novelty” from the child's perspective, failing to step outside the established framework of toys, tasks, and objectives. He was unable to adopt a more open attitude, understand the child's interests from his viewpoint, and engage in appropriate interactive behaviors.

#### 5.2.4 Sub-pathway 4 in free play

**Figure 5:**
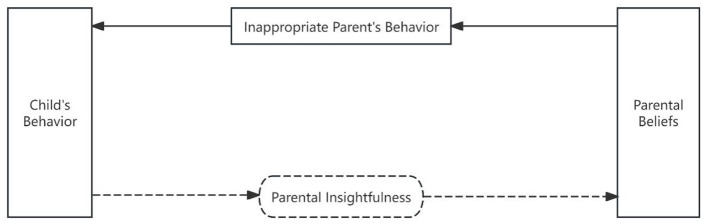
Sub-pathway 4 in free play.

Figure 5 presents that, within Sub-pathway 4 of Psychological Pathway Two, parents ignore children's signals and directly initiate or redirect interactions based solely on their parenting beliefs. There is a mismatch between parenting beliefs and children's needs, resulting in inappropriate interactive behaviors.

Vignette 4: Lele (female, 33 months) was building blocks. She tried to put three pieces in a row, end to end. Her mother (35 years) picked up a block and said, “Lele, Mommy got this block. What color is it?” Lele took a look and replied “green,” and then continued to play with her blocks. The mother pointed to the middle block and asked, “There's something here. What's in here?” Lele picked up the block her mother pointed to, murmured something to herself, looked back to the blocks she built, and then squeezed them from both ends.

Analysis: In the above recording, the child showed obvious interest in building blocks, persistently attempting to join them together (child's signal 1). During the interview, the mother mentioned: “Take advantage of playtime to ask questions about colors, shapes, and such-that way you can teach something along the way” (parent's view of education) The mother, however, interrupted the play by asking an irrelevant question (parent's behavior 1). After giving the mom an answer, the child immediately returned to her own play (child's signal 2). The mother apparently did not realize that her action had interfered with child's independent play because she asked another irrelevant question (parent's behavior 2). The mothers should patiently observe the points of interest in child's block-building activities. A teachable moment occurs only when the educational objective (learning block features) aligns with the child's signals (showing interest in block features).

#### 5.2.5 Sub-pathway 5 in daily caregiving

**Figure 6:**
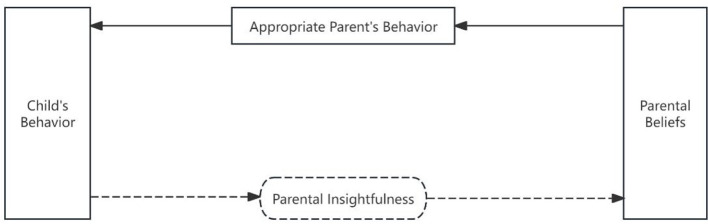
Sub-pathway 5 in daily caregiving.

Figure 6 presents that, within Sub-pathway 5 of Psychological Pathway Two, parents ignore children's signals and initiate or redirect interactions based solely on their parenting beliefs. However, their beliefs match children's current needs, resulting in appropriate interactive behaviors.

Vignette 5: Guoguo (female, 32 months) and her mother (30 years) pretended to brush teeth. The mother gave a tube of toothpaste to the child and instructed, “Squeeze the toothpaste yourself”. Guoguo did it with a smile and gave it back to her mother. The child then stood on a small stool and brushed her teeth in front of the sink while holding a cup. The mother said, “Wow, I hear the sound. Aren't there a lot of bubbles? Let me have a look. Wow, there are a lot of bubbles.” The child brushed her teeth and rinsed her mouth independently.

Analysis: The recording shows the mother's positive feedback, “I hear the sound” during the baby's toothbrushing process (parent's behavior 1). The child's brushing behavior was affirmed by the mother. Furthermore, “bubbles”, a word that most children adore, made brushing more enjoyable and fully sparked the child's enthusiasm (parent's behavior 2). She independently carried out the tasks of squeezing toothpaste, brushing teeth, and rinsing her mouth. During the interview, the mother indicated that the child was very reluctant to brush her teeth before. Her mother felt challenged, searched online for solutions, and found several tips. In her opinion, children only cared about eating, drinking, and playing, and she understood brushing teeth could be tricky and boring for children (parent's view of child). Therefore, by combining her own observations of the child's thoughts, the mother helped the child adapt to and enjoy the toothbrushing process by involving family members in brushing together and demonstrating how brushing creates bubbles (parent's behavior 3). She said she was open to her child's rejection of brushing teeth and never forced her to do so. She understood the tricky task from her child's perspective and modified her responses based on the child's preference. The mother's tolerant approach to parenting helped the child overcome the challenge of brushing teeth (parent's view of education).

## 6 Discussion

Inspired by dual-process models of information processing and Responsive Caregiving, this study constructs a “*Dual-Factor, Dual-Pathway Model*” hypothesis for parent–child interaction (Petty and Cacioppo, 1986; World Health Organization, 2018). Through quantitative and qualitative analysis, the validity of this model is verified. The dual factors are “parental insightfulness” and “parental beliefs,” while the dual pathways are the “Parental Insightful-ness-dominant Pathway” and the “Parental Belief-dominant Pathway.” The former is influenced by parental insightfulness, with the pathway's critical factor being parents' ability to consistently monitor children's signals, accurately interpret their motivations and needs, and apply this understanding to interaction direction and goals. The latter is influenced by parental childrearing beliefs, with the pathway's critical factor being whether these beliefs are accurate and aligned with the child's signals during the current interaction. This section will explore the two research questions posed in the introduction.

### 6.1 Parents favor parental insightfulness-driven pathway in parent–child interactions

Within Parental Insightfulness-driven Pathway, most parents (69.9%) were able to interpret children's signals accurately, provided appropriate interactive behaviors, and facilitated high-quality parent–child interactions. The finding highlights the positive impact of parents' sensitive attention to and accurate interpretation of the child's needs on interaction quality. High quality parent–child interactions consist of parents' close attention to children's verbal and non-verbal signals and appropriate responses to their needs in time (Kaiser et al., 1996). Our findings confirmed key traits of a responsive and healthy interaction, that is, sensitive attention, accurate interpretation, and appropriate response.

It is worth noting that some parents (29.1%) who displayed parental insightfulness also applied their own preconceived beliefs to guide the interactions as Sub-pathway 2 (27.1%) and Sub-pathway 3 (3.0%) indicate. These parents demonstrated commendable sensitivity and effort in perceiving and interpreting their children's signals, which reflects their attempts to understand and respond to their children's needs. However, they were still impacted by pre-conceived parenting beliefs, adopted unsuitable communication strategies, and consequently provided inappropriate responses. There are two possible explanations. Firstly, parent–child interactions often occur spontaneously and shift rapidly, leaving parents insufficient time to analyze the unique child behaviors and their underlying motives. In situations where children express no clear signals and parents lack patience to wait, they may quickly conduct interactions based on their own parenting beliefs (Lee, 2011). Secondly, some children provide ambiguous or inaccurate interactive signals, therefore parents are forced to use their preconceived beliefs to guide their interactions. This tendency reflects cognitive inertia and habitual thinking patterns inherent in human cognition (Colvin et al., 2021). Cognitive patterns prompt parents to interpret children's expressions and behaviors through pre-existing informational frameworks, often failing to adequately consider the specific context and the current unique situation (Beckerman et al., 2017). Our findings show some parents exhibited rigid cognitive concepts. Even though the children changed their expressions or behaviors, the parents still struggled to effectively update their judgments. Consequently, their schemas continued to shape their understanding and evaluation and underpinned their outdated interactive approaches.

### 6.2 When parents act from parental Insightfulness, they are more likely to engage in appropriate interactions

Parents showed a stronger preference for the Parental Insightfulness-dominant Pathway (59.6%) over the Parental Beliefs-dominant Pathway (40.4%), which indicates heightened sensitivity, openness, and the ability to accurately discern motivations. As revealed in interview data, these competencies are profoundly shaped by personal developmental experiences, reflective insights into one's own parenting practices, and deliberate lifelong learning.

First, we hypothesize that parents' personal developmental experiences profoundly influence their sensitivity, openness, and accurate recognition abilities in interactions. Parents who experienced a lack of emotional responsiveness or inconsistent caregiving in early life may exhibit avoidance, defensiveness, or controlling tendencies when confronted with children's emotional signals (George and Solomon, 2008). Conversely, it can be inferred that parents who experienced secure attachment and positive emotional responsiveness during childhood are less likely to exhibit these issues. Furthermore, the internal working models formed by parents' own developmental experiences influence how they interpret their child's signals, making them more likely to respond according to pre-existing parenting scripts rather than the immediate interaction context (Pietromonaco and Barrett, 2000). Personal developmental experiences not only shape parents' emotional expectations for parent–child relationships but also determine their response patterns to children's unexpected behaviors. Secure and integrated developmental experiences foster parental psychological resilience, enabling them to respond to children with understanding and acceptance during interactions. This fosters more sensitive and cooperative relationships (Slade, 2005). Second, reflective functioning refers to parents' ability to understand and infer children's internal psychological states and their relationship to overt behaviors (Slade, 2005), closely linked to their sensitive insight during interactions. Parents with higher reflective functioning recognize the gap between their own emotional reactions and children's signals during interactions, reducing automated responses based on past experiences and shifting toward sensitive responses centered on children's present needs. High reflective functioning prompts parents to recognize the potential multi-layered emotions and intentions underlying a child's behavior. This fosters an attitude of curiosity, acceptance, and exploration, rather than immediately interpreting the behavior through an adult lens as a “problem” or “defiance” (Luyten et al., 2017). This demonstrates that such mentalizing capacity enables parents to temporarily suspend automatic responses when confronted with unfamiliar or unexpected child behaviors. They engage in psychological inference and reinterpretation, responding to the child in a more open and flexible manner. Finally, parents' continuous active learning and accumulation of updated parenting experiences (Eldeeb et al., 2025) prompt them to transcend traditional or fixed parenting frameworks. This enables them to reinterpret the developmental significance behind children's behaviors, making them more inclined to interact based on the child's current signals.

### 6.3 Parents who base their parenting on their own parental beliefs are more likely to engage in inappropriate interactions

This study found that when parents used their parental beliefs, their interactive behaviors were often inappropriate. This was evidenced by 72.2% of the parents who used Psychological Pathway Two in the parent–child interactions, featuring misinterpretations of children's signals frequently led to inappropriate responses. Parents with rigid parenting beliefs often fail to accommodate the inherently flexible process of interactions. Consequently, few interactive behaviors stemming from such beliefs align with children's immediate interests and needs and are deemed appropriate. The findings were consistent with the top-down processing theories, which state that parents' prior knowledge guide their response (Petty and Cacioppo, 1986). Prior knowledge is always unable to address spontaneous and immediate needs of children during interactions. This is likely due to long held but false parenting notions. The evidence from our interview data supports such an assumption. For example, one participant stated:

“*When I'm with my son, I usually remain close. I don't take initiative if he doesn't need me. But when he needs me, I might give him some feedback. Most often, he plays alone.”*

This father revealed that he wanted to give his son sufficient freedom for independent exploration and intervened only when necessary. While his respect for the child's independence is commendable, he might have missed many valuable interaction moments. He only provided help when the child encountered difficulties and actively sought help. This scenario illustrates how parents' underlying views of raising children shape interactive responses and affect parent–child relationships. The finding was consistent with previous research indicating that parental expectations of their children influenced interactions, with worse parent–child connections being associated with high expectations of success and support for punishment (Ciciolla et al., 2017). Parents need to adapt and modify their parenting practices, integrate prior positive parenting experiences, if possible, to guide their interactions.

## 7 Limitations and implications

This study had two main limitations. Firstly, the age range (i.e., 12–36 months) of the toddlers was narrow. Their language skills were emerging, and this could constrain the verbal signals the parents could receive from them. Secondly, this study features a small sample size and exclusively encompasses Chinese parents, with a higher proportion of highly educated and high-income parents within the sample. This study had originally planned to recruit a larger sample size, but this proved unfeasible due to many parents declining to participate in video recordings, thereby limiting the general applicability of the findings. Subsequently, stratified sampling may be employed according to household socioeconomic status to expand the sample size and enhance representativeness. Thirdly, while the research subjects were all drawn from Chinese families, the analysis drew upon Western theoretical frameworks and lacked cross-cultural comparative samples. Future research may explore cross-cultural studies of insight preferences and implicit process model.

Despite these limitations, this study nevertheless holds value. Firstly, in terms of research content, while existing studies predominantly focus on behavioral characteristics within parent–child interactions, this research further examines the underlying implicit psychological processes. Specifically, it constructs an implicit process model to explain how parental insightfulness influences parent–child interactions. Secondly, in terms of methodology, this study adopts a micro-dynamic perspective, innovatively employing turn-by-turn analysis with each interaction turn as the unit of analysis. Building upon this, it develops a “*Dual-Factor, Dual-Pathway*” Framework, demonstrating considerable general applicability.

Our findings provide the following evidence-based implications. Firstly, parents should be aware of the importance of parental insightfulness in their interactions with their children, and if possible, training on enhancing parental insightfulness should be provided. Secondly, parents should observe children's verbal, behavioral, and emotional indicators and avoid imposing their possibly incorrect views, beliefs, and overwhelming expectations on children. Thirdly, parents should be proactive in learning high-quality interaction techniques. Finally, parents should constantly reflect on interactions with their children and continuously refine their interpretation and responsive strategies in light of children's feedback, in order to improve the quality of interactions and further enhance their relationships with the children.

## Data Availability

The datasets presented in this article are not readily available because the authors do not have permission to share data. Requests to access the datasets should be directed to the corresponding author.
